# High Incidence of Copy Number Variants in Adults with Intellectual Disability and Co-morbid Psychiatric Disorders

**DOI:** 10.1007/s10519-018-9902-6

**Published:** 2018-06-07

**Authors:** Marina Viñas-Jornet, Susanna Esteba-Castillo, Neus Baena, Núria Ribas-Vidal, Anna Ruiz, David Torrents-Rodas, Elisabeth Gabau, Elisabet Vilella, Lourdes Martorell, Lluís Armengol, Ramon Novell, Míriam Guitart

**Affiliations:** 1 Genetics lab, UDIAT-centre diagnostic. Parc Taulí Hospital Universitari. Institut d’Investigació i Innovació Parc Taulí I3PT. Universitat Autònoma de Barcelona, C/Parc Tauli,1, 08208 Sabadell, Barcelona Spain; 2 Cellular Biology, Physiology and Immunology Department, Universitat Autònoma de Barcelona, Bellaterra, Barcelona, Spain; 3 Mental Health and Intellectual Disability Specialized Service, Institut Assistència Sanitària (IAS), Parc Hospitalari Martí i Julià, Girona, Spain; 4 Pediatry-Clinical Genetics Service, Parc Taulí Hospital Universitari. Institut d’Investigació i Innovació Parc Taulí I3PT. Universitat Autònoma de Barcelona, Sabadell, Spain; 5 Hospital Universitari Institut Pere Mata, IISPV, Universitat Rovira i Virgili, CIBERSAM, Reus, Spain; 6 Research and Development Department, qGenomics Laboratory, Barcelona, Spain

**Keywords:** Adult patients, Behavioural disorders, Copy number variants, Intellectual disability, Psychiatric disorders

## Abstract

A genetic analysis of unexplained mild-moderate intellectual disability and co-morbid psychiatric or behavioural disorders is not systematically conducted in adults. A cohort of 100 adult patients affected by both phenotypes were analysed in order to identify the presence of copy number variants (CNVs) responsible for their condition identifying a yield of 12.8% of pathogenic CNVs (19% when including clinically recognizable microdeletion syndromes). Moreover, there is a detailed clinical description of an additional 11% of the patients harbouring possible pathogenic CNVs—including a 7q31 deletion (*IMMP2L*) in two unrelated patients and duplications in 3q29, 9p24.2p24.1 and 15q14q15.1—providing new evidence of its contribution to the phenotype. This study adds further proof of including chromosomal microarray analysis (CMA) as a mandatory test to improve the diagnosis in the adult patients in psychiatric services.

**Electronic supplementary material:**

The online version of this article (10.1007/s10519-018-9902-6) contains supplementary material, which is available to authorized users.

## Introduction

Intellectual disability (ID) is a complex and multifactorial disorder that includes both intellectual and adaptive functioning deficits in the conceptual, social and practical domains with onset during the developmental period. This disorder affects approximately 1–3% of the general population, and between 10 and 40% of people with ID also present with mental illness or behavioural disorders (Cooper et al. 2007; Lowe et al. 2007; Morgan et al. 2008). The diagnostic categories of these mental disorders are based on the symptoms (Stein et al. 2013), but there is considerable clinical heterogeneity and overlap with different psychiatric categories (Burmeister et al. 2008). Indeed, the boundaries of the diagnostic categories can be blurred when the patients’ symptoms are not clearly expressed. The diagnosis of a psychiatric disorder in subjects with ID can be difficult, and most symptoms tend to be attributed to the ID. For this reason, the co-occurrence of both entities is usually overlooked (Costello and Bouras 2006).

Copy number variants (CNVs) are a source of human genetic variation and have been described as an important genomic cause of human disease (Iafrate et al. 2004; Sebat et al. 2004). Screening of ID patient cohorts via chromosomal microarray analysis (CMA) has led to the characterization of new syndromes, such as 8q21.11 deletion syndrome (OMIM: 614230) and 19p13.3 microdeletion/microduplication syndrome (Dolan et al. 2010; Orellana et al. 2015). Additionally, there is evidence that CNVs can predispose individuals to the development of psychiatric disorders, such as the autism spectrum disorders (ASDs) (Marshall et al. 2008; Hedges et al. 2012), schizophrenia (SQZ) (Kirov et al. 2012; Xu et al. 2008), bipolar disorder (Green et al. 2015) and attention-deficit/hyperactive disorder (ADHD) (Jarick et al. 2014; Ramos-Quiroga et al. 2014). Numerous CNV loci have been recurrently observed across ID and various neuropsychiatric phenotypes, such as the16p11.2 and *NRXN1* deletions, both of which are associated with ID, SQZ and ASD. These findings suggest that ID and psychiatric disorders may share genetic susceptibility factors (Guilmatre et al. 2009).

A large proportion of the adult population affected by ID lacks a genetic diagnosis. Some of these adult patients have never received a diagnostic assessment; alternatively, in some cases the assessment is completed without finding an explanation for the ID possibly due to the use of less advanced technologies than are currently available. At present, there is little knowledge of the genetics of ID and co-morbid psychiatric disorder in adults. Nevertheless, CMA and whole exome sequencing could shed light on the genetic diagnoses in adults with idiopathic ID (Baker et al. 2012; Posey et al. 2016; Taylor et al. 2010; Wolfe et al. 2016). Here, we report the genetic analysis of 100 adult patients affected by ID and psychiatric and/or behavioural disorders. The main purpose of this study is to investigate the contribution of putative pathogenic CNVs among patients with ID and comorbid psychiatric/behavioural disorders.

## Materials and methods

### Participants

This study was designed prospectively. Cognitive, psychiatric and behavioural evaluation was performed by psychiatric specialists at the Mental Health ID Service (“Parc Hospitalari Martí i Julià”, Girona, Catalonia, Spain) while clinical-dysmorphic evaluation and genetic assessment was performed by a clinical geneticist at the Clinical Genetics Department (“Parc Taulí Hospital Universitari”, Sabadell, Catalonia, Spain). This study was approved by the institutional ethics committee (CEIC 2009/582). A legal guardian or family member that legally represented the participant signed the informed consent form. Adult patients over the age of 18 years were consecutively recruited using the following inclusion criteria: mild (IQ = 75 − 50) or moderate (IQ = 50 − 35) ID according to the Diagnostic and Statistical Manual of Mental Disorders (DSM-5) criteria and a defined psychiatric disorder or behavioural disorder according to the measures listed in Table 1. The exclusion criteria were having severe ID or sensory impairment that precluded a proper examination, having suffered alterations in the central nervous system unrelated to the ID (i.e., head injury, stroke or brain tumours), the presence of untreated diseases with associated cognitive deficits (i.e., hypothyroidism, vitamin B12 deficiency or diabetes mellitus) and substance abuse. This recruitment led to 100 eligible patients for the analysis, including five sibling pairs and a sibling trio.

**Table 1:** Cognitive, psychiatric and behavioural measures tests

Measures	Tests
Cognitive	– K-BIT-II (Kauffman Brief Intelligence Test-II) – ABS-RC2 first part (Adaptive Behaviour Scale Residence Community-2)
Psychiatric	– PAS-ADD (Psychiatric Assessment for Adults with Developmental Disabilities) – Compulsive behaviour checklist – Y-BOCS (Yale-Brown Obsessive Compulsive Scale) – RBQ (Repetitive Behaviour Questionnaire) – NPI (Neuropsychiatric Inventory)
Behavioural	– ABC-ECA Scale (Aberrant Behaviour Checklist) – ABS-RC2 second part (Adaptive Behaviour Scale Residence Community-2)

### Clinical evaluation: cognitive, behavioural, psychiatric and dysmorphic measures

Different tests were administered to all participants to identify the presence of ID and establish its severity level, as well as to identify the presence of a psychiatric and/or behavioural disorder (Table 1). The presence of a behavioural disorder not necessarily related to a mental disorder was defined according to (Emerson 1995) as “culturally abnormal behaviour of such intensity, frequency or duration that the physical safety of the person or others is placed in serious jeopardy, or behaviour which is likely to seriously limit or deny them access to ordinary community facilities”. A family history of ID, psychiatric or behavioural disorders was also recorded.

Dysmorphic features were classified into five categories as follows: craniofacial, limbs, cutaneous, genital and body (all other dysmorphisms). A category was considered dysmorphic if at least one feature was abnormal.

### Genetic analysis

The cohort was first analysed by G-banded karyotyping to determine the presence of unbalanced and balanced rearrangements. *FMR1* screening and other specific molecular technologies were applied to subjects who were clinically suspected of having a syndrome.

The CMA analysis was performed with the 400K Agilent platform (Agilent Technologies, Santa Cruz, CA, USA) on all patients without a clinically recognized syndrome (including subjects known to possess a chromosomal rearrangement). This oligonucleotide-based comparative genomic hybridization array covered the entire genome with an average resolution of 5.3 kb. The microarrays were processed according to the manufacturer’s specifications, and the Agilent Workbench 5.0, Feature Extraction and Cytogenomics softwares (Agilent Technologies, Santa Cruz, CA, USA) were used to render the image analysis with the manufacturer’s recommended settings and human genome assembly hg19. We called CNVs when there were at least five consecutive probes with a minimum log_2_ ratio of ± 0.25.This low rate is capable to detect mosaicisms and using five consecutive probes avoid false positives.

The identified CNVs were cross-referenced with the Database of Genomic Variants (DGV, http://projects.tcag.ca/variation); those variants completely overlapped with common CNVs (prevalence > 1% in the general population) were excluded from further analysis. All rare CNVs (prevalence < 1% in the general population) were interpreted individually by comparing each genomic region to information available in public databases [University of California, Santa Cruz Genome Browser (http://genome.ucsc.edu), National Center for Biotechnology Information (http://www.ncbi.nlm.nih.gov), Ensembl (http://www.ensembl.org/index.html), Decipher (https://decipher.sanger.ac.uk/), Clinical Genome Resource (https://www.clinicalgenome.org)] and Online Mendelian Inheritance in Man database (https://www.omim.org/) as well as literature, and classified into four categories as follows: (1) Pathogenic CNVs (pCNV), which overlap with known causative findings previously associated with ID or psychiatric disorders (from databases and literature). (2) Variants of unknown significance (VOUS) that were likely pathogenic (pVOUS) when at least two of the following conditions are met: (a) Partially overlap with a pathogenic susceptibility *locus*; (b) It is not reported in control population from (Coe et al. 2014); (c) Include genes enriched for deletions/duplications at nominal level of significance according to (Coe et al. 2014); (d) Include developmental delay (DD) genes from (Deciphering Developmental Disorders Study 2017); (e) Include genes with relevant function in the nervous system. (3) VOUS that were likely benign (bVOUS), which included only intronic regions of genes with a function in the nervous system not yet described in the patients or CNVs that included genes with unknown functions or functions not related to the central nervous system. (4) Benign CNVs (bCNVs), which were without genes or devoid of known regulatory elements. We focused on pCNVs and pVOUSs, both of which are likely associated with the affected phenotype. Customized multiplex ligation-dependent probe amplification (MLPA) and fluorescent in situ hybridization (FISH) were performed according to standard protocols to validate and determine the inheritance of CNVs. Custom MLPA probes were designed according to protocols and guidelines from MRC-Holland (Amsterdam, the Netherlands) and the ProSeek web server created by Estivill et al. (Pantano et al. 2008), and specific bacterial artificial chromosome clones were selected for the aberration regions.

Finally, since the cohort of 100 patients results from a 50-patient set which was subsequently increased with a second 50-patient set, we selected seven CNVs (pCNVs and pVOUS) identified in the first patient-set analysed by CMA to evaluate their recurrence. Two pCNVs associated with ID and psychiatric disorders (2p16.3 and 12p12.1) and five pVOUSs (2p12, 3q29, 15q14q15, 15q26.2 and 17q24) were analysed in a new set of 161 adult patients affected by mild/moderate ID and 189 controls using a custom MLPA.

### Data analysis

The potential associations between categorical variables were tested using the *χ*^*2*^ test. When one or more of the expected values for the *χ*^*2*^ computation was lower than 5, the *p* value was computed using Fisher’s exact test. When a result was significant, the odds ratio was indicated as a measure of the effect size. The Kruskal–Wallis and Mann–Whitney *U* tests were performed for dependent continuous variables that showed non-normal distributions (as determined by the Shapiro–Wilk test and visual inspection). A threshold of p < 0.05 was set to indicate statistical significance, and the Bonferroni correction was applied for post hoc comparisons. The statistical analyses were performed using the Statistical Package for Social Sciences (SPSS for Windows, Version 16.0., SPSS Inc., Chicago, IL, USA).

## Results

### Description of the patient cohort

A patient cohort of 100 adults affected by ID and co-morbid psychiatric/behavioural disorders without a genetic diagnosis was recruited with the main purpose of identifying CNVs responsible for their conditions. The cohort comprised 50 men and 50 women with an average age of 31.28 years (18–56 years, SD = 10.14), of whom 60% had mild ID and 40% had moderate ID. Out of the 100 patients, 50 had both a psychiatric and a behavioural disorder, 37 had only a psychiatric disorder and 13 had only a behavioural disorder. Sixteen patients had a diagnosis of two different psychiatric disorders, nine of whom also presented with a behavioural disorder. Table 2 shows the distribution of the psychiatric disorders in our cohort according to ID severity level and the presence or absence of behavioural disorders. The *χ*^*2*^ test did not show a significant difference in the presence of psychiatric or behavioural disorders between the mild and moderate ID groups. Mild dysmorphic features were present in all patients and were identified via minor facial or cranial dysmorphologies (98%) and abnormalities in the limbs (44%), cutaneous tissue (52), genitals (16%) and other (60%).

**Table 2:** Distribution of the psychiatric disorders according to the ID severity level and the presence or absence of behavioural disorders

Psychiatric disorders (n = 116)*	Mild ID	Moderate ID
With behavioural disorders	n = 36	n = 36
Organic mental disorders (F01–F09)	2 (5.6%)	0
Schizophrenia spectrum (F20–F29)	3 (8.3%)	1 (2.8%)
Depressive disorders (F30–F39)	3 (8.3%)	0
Anxiety (F40–F48)	14 (38.9%)	12 (33.3%)
Non-organic disorder of the sleep-wake schedule (F51.2)	0	0
Personality disorders (F60–F69)	7 (19.4%)	3 (8.3%)
Psychological developmental disorders (F80–F89)	0	3 (8.3%)
Childhood behavioural/emotional disorders (F90–F98)	3 (8.3%)	8 (22.2%)
No diagnosable disorder	4 (11.1%)	9 (25%)
Without behavioural disorders	n = 33	n = 11
Organic mental disorders (F01–F09)	0	0
Schizophrenia spectrum (F20–F29)	5 (15.2%)	3 (27.3%)
Depressive disorders (F30–F39)	7 (21.2%)	1 (9.1%)
Anxiety (F40–F48)	15 (45.5%)	2 (18.2%)
Non-organic disorder of the sleep-wake schedule (F51.2)	0	1 (9.1%)
Personality disorders (F60–F69)	1 (3%)	0
Psychological developmental disorders (F80–F89)	3 (9.1%)	3 (27.3%)
Childhood behavioural/emotional disorders (F90–F98)	2 (6.1%)	1 (9.1%)
No diagnosable disorder	0	0

*The table includes the 116 psychiatric diagnoses identified in the adult cohort (n): 36 in patients with mild ID and behaviour disorders; 36 in patients with moderate ID and behaviour disorders; 33 in patients with mild ID without behavioural disorders; 11 in patients with moderate ID without behavioural disorders. There were 16 individuals with two different psychiatric disorders

### Genetic analysis of the patient cohort

A preliminary karyotype identified four rearrangements and the specific molecular technologies confirmed the presence of a clinically recognized syndromes in fourteen individuals (Table 3). The CMA performed in the 86 patients with no clinically recognized syndromes identified a total of 216 rare CNVs (additional file 1) with an average of 2.5 CNVs/patient and range of 0–8 CNVs/patient. According to the classification criteria, 13 pCNVs were the genetic cause of the phenotype and 11 pVOUSs were the putative cause of the phenotype (additional file 2) while 192 CNVs (88.9%) were non-pathogenic (139bVOUSs and 53 bCNVs). The 13pCNVs, nine deletions and four duplications, were identified in 11 of the 86 patients (12.8%) (Table 4), given that two patients presented two CNVs—in one case the 2 pCNVs arose from a maternal inversion (patient 10) and in the other case the 2 pCNVs derived from an unbalanced translocation (patient 26) according to the FISH performed afterwards. The 11pVOUS, five deletions and six duplications, were identified in 11 of the 86 patients (12.8%) (Table 4), but if we consider only one patient of each sibling set (given that we include four set of siblings in the CMA population), pVOUS are the putative cause of disease in nine of 82 patients (11%). The analysis of parental samples (when available) revealed that the pCNVs were de novo in seven patients and maternally inherited in two cases (one X-linked). In contrast, of the eight cases with pVOUSs with available parental samples, there were no de novo pVOUSs (Table 4).

**Table 3:** Well-known specific syndromes

Syndrome	Genetic cause	No. cases
Fragile X	CGG expansion	5
Velocardiofacial	22q11.2 deletion	4
Prader Willi	15q11q13 deletion	2
Smith Magenis	*RAI1* point muation	1
	17p11.2 deletion	1
Williams	7q11.23 deletion	1

**Table 4:** Phenotypic and genotypic description of patients with pathogenic and likely pathogenic CNVs

Pat Id	ID		Psychiatric disorder	Behavioural disorder		Dysmorphology	FHD		ISCN 2016	CNV size (kb)	RefSeq genes
Pathogenic CNVs (pCNV)											
55 ♀	Mild		BD	NP		Hypotelorism, high and narrow palate		+	arr[hg19] 2p16.3(50660882–51078593)x1 dn	417	*NRXN1*
94 ♂	Mild		Persistent delusional disorders	Verbally aggressive, physically aggressive and destructive behaviours		Long face, wide forehead, long philtrum, high and narrow palate		+	arr[hg19] 2p16.3(50510602–51137271)x1 mat	626	*NRXN1*
90 ♂	Mod		Specific (isolated) phobias; Adjustment disorders with mixed disturbance or emotions and conduct	Verbally aggressive, oppositional, demanding and other problem behaviours		Brachycephaly, long face, synophrys, blepharophimosis, downslanted palpebral fissures, short ears, prognathism, kyphosis, absent distal interphalangeal creases, generalized hirsutism		+	arr[hg19] 9q31.1q32(107056010–115867141)x1	8,811	67
26 ♂	Mod		OCD ADHD	Physically aggressive behaviour		Long face, strabismus, broad nasal tip, dysplastic ears, high and narrow palate, widely-spaced nipples		–	arr[hg19] 10q26.12q26.3(122259702–135434178)x1 der	13,174	113
									arr[hg19] 15q26.3(99168589–102480888)x3 der	3,312	32
63 ♀	Mild		NP	General diagnostic criteria for problem behaviour		Narrow nasal bridge, broad nasal tip, high and narrow palate, retrognathia, generalized hirsutism		–	arr[hg19] 12p12.1(23432294–26233996)x1 dn	2,801	14 (*SOX5*)
98 ♂	Mild		Specific (isolated) phobias	Physically aggressive behaviour		Macrocephaly, strabismus, high and narrow palate		–	arr[hg19] 15q11q13(23,699,701–29,006,852)x3 dn	5,306	126
14 ♂	Mild		Generalized anxiety disorder	NP		High and narrow palate, dental malocclusion, obesity, gynecomastia, generalized hirsutism, macroorchidism		+	arr[hg19] 15q13.2q13.3(30943703–32439084)x1 dn	1,495	10 (*CHRNA7*)
9 ♀	Mod		Acute polymorphic psychotic disorder without symptoms of schizophrenia	NP		Microcephaly, low posterior hairline, strabismus, small nose, wide nasal base, prognathism, short stature, scoliosis, nasal voice		–	arr[hg19] 16p12p11.2(18901309–29182196)x1 dn ^(a)^	10,280	131
18 ♀	Mod		Childhood ASD	Physically aggressive, destructive, oppositional and other problem behaviours		Puffy eyelids, broad nasal bridge, downslanted palpebral fissures, high and narrow palate		–	arr[hg19] 22q13.33(51123291–51224402)x1 dn	101	4 (*SHANK3*)
71 ♂	Mild		Other organic personality and behavioural disorders	Verbally aggressive, physically aggressive and oppositional behaviours		Round face, thick lower lip vermilion, dental malocclusion, obesity, flat feet		+	arr[hg19] Xp24.3p11.4(25816432–38085678)x2 mat ^(b)^	12,244	38
10 ♀	Mod		NP	Physically aggressive and oppositional behaviours		Macrocephaly, long face, wide nasal base, broad philtrum, macrostomia, high and narrow palate, gingival overgrowth, Sydney crease, camptodactyly, abnormal labia, capillary hemangioma		+	arr[hg19] Xp22.33p11.2(169901-51101339)x3 mat inv ^(c)^	51,008	360
									arr[hg19] Xq25q28(124,642,297–155,227,312)x1 mat inv ^(c)^	30,410	335
VOUS likely pathogenic (pVOUS)											
34 ♂	Mild		Post-traumatic stress disorder	Verbally aggressive, physically aggressive and wandering behaviours		Epicanthus, strabismus, thin upper lip, high and narrow palate, dental malocclusion, obesity, gynecomastia		+	arr[hg19] 3q29(196,022,728–196,515,371)x4 mat-pat	492	14
32 ♂	Mod		Post-traumatic stress disorder	NP		Turricephaly, long face, accessory nipples		+	arr[hg19] 7q31.1(111198987–111280493)x1 mat	81	*IMMP2L*
151 ♂	Mod		OCD; Childhood ASD	Verbally aggressive and physically aggressive behaviours		Sloping forehead, strabismus, fullness of upper eyelid, bilateral preauricular pit, protruding ears and underdeveloped crus of the helix, wide nasal base, everted lower lip vermilion, widely spaced teeth, cryptorchidism		+	arr[hg19] 7q31.1(111112186–111255558)x1 mat	143	*IMMP2L*
66 ♂	Mild		Specific (isolated) phobias; Generalized anxiety disorder	NP		Long face, synophrys, hypotelorism, thin upper lip, broad jaw and prognathism		+	arr[hg19] 8q21.13(80288192–81019201)x1 pat	731	6
85 ♂	Mild		Dual-role Transvestism	Verbally aggressive, destructive, sexually inappropriate, oppositional, demanding and other problem behaviours		Microcephaly, long face, narrow forehead, Low hanging columella, dental malocclusion, small testes		–	arr[hg19] 8p23.1(10254051–10449952)x1	195	3
122 ♂ ^(*)^	Mod		NP	Verbally aggressive behaviour		Ptosis, long and protruding ears, broad and bifid nasal tip, large tongue, pectus excavatum, macroorchidism		+	arr[hg19] 9p24.2p24.1(4094627–4671089)x3	576	4
123 ♂ ^(*)^	Mod		Generalized anxiety disorder	Verbally aggressive and physically aggressive behaviours		Macrocephaly, long face, broad forehead and metopic depression, protruding and low-set ears, long philtrum, thin upper lip, high and narrow palate		+	arr[hg19] 9p24.2p24.1(4094627–4671089)x3	576	4
59 ♀ ^(#)^	Mod		ADHD	Verbally aggressive, physically aggressive, destructive, oppositional, demanding and wandering behaviours		Long face, hypotelorism, epicanthus, ptosis, broad jaw, long fingers		+	arr[hg19] 15q14q15.1(37882913–40621860)x3 pat	2,738	20 (*SPRED1*)
60 ♂ ^(#)^	Mod		OCD; ADHD	Sexually inappropriate, demanding and wandering behaviours		Microcephaly, hypotelorism, long face, ptosis, broad jaw, long fingers, tall stature, pectus excavatum, numerous pigmented freckles		+	arr[hg19] 15q14q15.1(37882913–40621860)x3 pat	2,738	20 (*SPRED1*)
92 ♂	Mild		Asperger’s Syndrome; Moderate depressive episode	NP		High and narrow palate		+	arr[hg19] 15q26.2(94959126–94983622)x1 pat	24	*MCTP2*
79 ♂	Mild		Acute stress reaction; Other habit and impulse disorders	Physically aggressive behaviour		Long face, long ears and large earlobe, thin upper lip vermilion, exaggerated cupid’s bow, high and narrow palate		+	arr[hg19] 17q24.1q24.2(64129644–64759936)x3 pat	630	4

*CNV* copy number variant, *Pat Id* patient identification; *ID* intellectual disability, *Mod* moderate, *BD* bipolar disorder, *OCD* obsessive–compulsive disorder, *ADHD* attention-deficit and hyperactive disorder, *ASD* autism spectrum disorder, *NP* not present, *FHD* familial history of psychiatric disorders; (+) Yes; (−) No; (mat inv) CNV derive from a maternal inversion; (der) derivative from a balanced translocation; (m‘at-pat) Inherited from both parents; (a) CNV identified by G-banded karyotype: 46,XX, del16p11.2p12; (b) CNV identified by G-banded karyotype: 46,XY,dup(X)(p11.4p24.3); (c) CNV identified by G-banded karyotype: 46,XX,der(X)inv(X)(p11.2q25),dup(X)(p11.2p22.33),del(X)(q25q28). (*) Siblings. (#) Siblings

Two shared CNV regions were present in unrelated patients. The first CNV region was the pathogenic 2p16.3 deletion in patients 55 and 94, which partially included the *NRXN1* gene (Table 4). The shared phenotype between these patients and the neuropsychological evaluation of deletion family carriers was previously reported (Vinas-Jornet et al. 2014). The second shared CNV was a 7q31.1 deletion that disrupted the *IMMP2L* gene, which encodes a catalytic subunit of the mitochondrial inner membrane peptidase (IMP) complex. This CNV was identified in two males (patients 32 and 151 from Table 4) affected by moderate ID and psychiatric disorders [a post-traumatic stress disorder in one patient and obsessive–compulsive disorder (OCD) with childhood autism in the other patient]. The deletion was maternally inherited in these two unrelated patients, and both patients had a registered familial history: patient 32’s mother was diagnosed with early Alzheimer’s disease and patient 151’s maternal aunt was diagnosed with a psychiatric disorder.

Family studies may help to understand the pathogenicity of CNVs and delineate genotype-phenotype correlations. Of the five sibling pairs included in the cohort, we identified a putative genetic cause that was shared between siblings in two pairs. A 9p24.2p24.1 duplication was identified in two brothers affected by moderate ID and behavioural disorders (patients 122 and 123), but a generalized anxiety disorder was diagnosed in only one patient (Table 4). This duplication overlaps two duplications described in DECIPHER in patients affected by cognitive and behavioural disorders (295,026 and 254,714, respectively). The second sibling pair was a female and her brother (patients 59 and 60) who were both affected by moderate ID, hyperkinetic conduct disorder and minor facial/cranial dysmorphology; the siblings shared a 2.7 Mb duplication in 15q14q15.1 (Table 4). There are overlapping duplications in public databases (ClinVar) with unknown clinical significance in patients with global developmental delay (nssv580863 and nssv1609978), the first of whom also presented with microcephaly and upslanted palpebral fissures.

Finally, a homozygous 3q29 duplication was identified in patient 34, who was affected by mild ID, post-traumatic stress disorder and behavioural disorders (Table 4). The patient was the third child of a consanguineous couple; both parents had borderline IQs and a heterozygous 3q29 duplication. The patient had a younger brother with a severe ID and ASD phenotype who also presented with the duplication in homozygosity.

### Effect of CNVs on dysmorphic and neurodevelopmental traits

Demographic and clinical variables (gender, ID severity level, dysmorphology, psychiatric disorders, behavioural disorders and psychiatric co-morbidity) from the group of patients with an identified putative genetic cause (pCNV and pVOUS) were compared to the patients with an unknown possibly genetic cause (bVOUS, bCNVs and absence of rare CNVs). The comparison of the number of dysmorphic features, the ID severity level, psychiatric disorders or behavioural disorders between the two groups did not show any significant difference. Interestingly, the odds of having two psychiatric disorders diagnosed in the same patient were 4.22 times higher in the genetic cause group than in the unknown possible genetic cause group (95% CI 1.21–14.74, *χ*^*2*^ (1) = 5.56, p = 0.035).

### Analysis of specific CNVs in an additional cohort

In order to evaluate the recurrence of seven pCNVs/pVOUS, an independent population of 161 patients affected by mild/moderate ID and 189 controls were analysed. None of the selected seven CNVs were detected either in the patients or the control individuals indicating a very low frequency of these pCNVs/pVOUS.

## Discussion

A genetic cause of the ID and psychiatric phenotypes was identified in 25 patients of our adult cohort. This incidence is due to the diagnosis of clinically recognized syndromes such as fragile X, Velocardiofacial, Prader Willi, Smith Magenis and Williams not recognised at the adult psychiatric service. The application of CMA test in those patients without a recognised syndrome allows the genetic diagnosis in 12.8% in agreement to a similar adult population affected by ID and co-morbid psychiatric disorders (Wolfe et al. 2016). This rate would have increased to 19% if the CMA had been performed in all patients being comparable to an adult population with ID and mild-severe congenital malformation anomalies (Ho et al. 2016).

Interestingly, in our series we found a *NRXN1* deletion in two cases responsible for bipolar disorder, persistent delusional disorders and behavioural phenotype (Vinas-Jornet et al. 2014) in keeping with (Lowther et al. 2017).

An additional 11% of the patients present pVOUS that may contribute to the phenotype despite there not being strong evidence for their pathogenicity. Given the low frequency of each individual CNV it is important to report them to increase the knowledge and clarify their possible association with the phenotype.

The 7q31 deletion identified in two unrelated patients (Table 4) disrupted the *IMMP2L* gene (NM_001244606). Although deletions in this region are reported as benign loss in ISCA database and are identified in control population, they are considered rare CNVs because their frequency is lower than 1% considering the DGV Gold Standard Variants (additional file 3). The 7q31 deletion was considered a risk factor in several neuropsychiatric disorders, including ASD (Maestrini et al. 2010; Pagnamenta et al. 2010; Casey et al. 2012), ADHD (Elia et al. 2010) and language disorder (Lai et al. 2001) and partial deletions of the *IMMP2L* gene in particular has been described as risk factors for neurological diseases with an incomplete penetrance (Gimelli et al. 2014). The history of Alzheimer’s disease in the carrier mother is interesting, particularly because the *IMMP2L* gene encodes a mitochondrial protein that regulates the levels of reactive oxygen species (George et al. 2011), and has been implicated in Alzheimer’s disease susceptibility (Swaminathan et al. 2012). This evidence suggests that *IMMP2L* may contribute to the ID and psychiatric disorders in these patients.

Little is known about the clinical effects of duplications in 3q29, 9p24.2p24.1 and 15q14q15.1 in contrast to the deletions in these regions that have been previously associated with neurodevelopmental disorders (Myles-Worsley et al. 2013; Bianchi et al. 2014; Spencer et al. 2011; Willatt et al. 2005). However, patients presented here suggest that these duplications could be pathogenic. The 3q29 duplication not only could disrupt the *PAK2* gene, which codifies a serine/threonine protein kinase involved in the dendritic development of early cortical neurons, but also includes the *FBXO45* gene. This gene, which is a component of an E3 ubiquitin ligase complex, is evolutionarily conserved and selectively expressed in the nervous system, plays an important role in the regulation of neurotransmission (Tada et al. 2010) and has been described as a candidate gene for SQZ (Wang et al. 2014). This CNV partially overlaps the 3.5 Mb critical region in 3q29 present in five members of a family affected by ID and microcephaly (Lisi et al. 2008) and spans some smaller duplications described in patients affected by ID and a wide range of minor dysmorphic features (Ballif et al. 2008). In our case, phenotypic severity correlated with the copy number of the 3q29 region in the proband, who harbours four copies of the 3q29 material and was affected by ID and a psychiatric disorder, and the parents, both of whom harbour three copies of 3q29 and had borderline IQs. A second putative pathogenic duplication identified in our cohort is located in 9p24.2p24.1 and includes the *SLC1A1* gene. This gene encodes a member of the high-affinity glutamate transporters, which are crucial for the termination of the postsynaptic action of the neurotransmitter glutamate and maintenance of extracellular glutamate concentrations below the neurotoxic levels. Changes in its expression are associated with neuropsychiatric diseases, such as OCD and SQZ (Porton et al. 2013; Bauer et al. 2008), and overexpression of *SLC1A1* has been demonstrated to increase the expression level of the two glial members of the glutamate transporter family (*SLC1A2* and *SLC1A3*), which are associated with SQZ (Afshari et al. 2015). Finally, although pathogenicity of the 15q14q15.1 duplication has not been demonstrated, this duplication includes three genes (*SPRED1, RASGRP1* and *PAK6*) that have been previously related to neuropsychiatric diseases (Brems et al. 2007; Denayer et al. 2008; Kato et al. 2011; Furnari et al. 2013). The presence of *SPRED1* is particularly interesting given that deletions and point mutations in this gene are responsible for Legius syndrome, which is a genetic skin pigmentation disorder that is sometimes accompanied by other common manifestations, including moderate ID, ADHD, hypotelorism and *pectus excavatum*; these symptoms were present in the two patients with the 15q14q15.1 duplication. This evidence suggests that the *SPRED1* gene may be responsible for the ID and neuropsychiatric disorders in our patients and that increased dosage in this region is capable of yielding a similar phenotype as decreased dosage.

Of the 13pCNVs and 11 pVOUSs, ten genes (*NRXN1, IMMP2L, MSRA, SLC1A1, SOX5, UBE3A, CHRNA7, SPRED1, PRKCA*, and *SHANK3*) have each been associated with more than one psychiatric phenotype (Table 5) and neurodevelopmental disorders based on the hypothesis that perturbation of the same molecular pathway can result in different psychiatric diagnoses (Plummer et al. 2016); for instance, *SHANK3* and *SLC1A1* participate in the glutamatergic pathway and *UBE3A* and *FBXO45* in the ubiquitin pathway (Javitt 2007, Tebartz van Elst et al. 2014; Glessner et al. 2009; Plummer et al. 2016). Other genes involved in synaptic formation and function may contribute to behaviour impairments and a brain malfunction (Mehregan et al. 2016). Interestingly, we found that the presence of two psychiatric disorders increases the likelihood of detecting a pathogenic or possibly pathogenic CNV supporting the fact that different psychiatric disorders share common genetic aetiologies (Moreno-De-Luca et al. 2013).

**Table 5:** Genes associated with various psychiatric disorders

Gene	Loci	Psychiatric disorder in current study	Bibliography*						
			ASD	ADHD	SQZ	BD	OCD	A	GTS
*NRXN1*	2p16.3	BD	+	+	+	+	–	+	+
		Delusional disorder							
		Disexecutive syndrome							
		Anxiety							
*IMMP2L*	7q31	GTS	+	+	–	+	+	–	+
		Post-traumatic stress disorder							
		OCD + autism							
*MSRA*	8p23.1	Transvestism, destructive and aggressive behaviour	–	–	+	+	–	–	–
*SLC1A1*	9p24.2p24.1	Generalized anxiety disorder	–	–	+	+	+	–	–
*SOX5*	12p12	Behavioural disorder	+	+	–	–	–	+	–
*UBE3A*	15q11q13	Specific phobias	+	+	+	–	+	+	–
*CHRNA7*	15q13.3	Generalized anxiety disorder	+	+	+	+	–	–	–
*SPRED1*	15q14q15	OCD	+	+	–	–	–	–	–
		Hyperkinetic disorder							
*RASGRP1*	15q14q15	OCD	–	–	–	+	–	–	–
		Hyperkinetic disorder							
*MCTP2*	15q26.2	Asperger syndrome	–	–	+	–	–	–	–
		Depressive episode with somatic syndrome							
*PRKCA*	17q24.1q24.2	Acute stress reaction	–	–	+	+	–	–	
		Other habit and impulse disorders							
*SHANK3*	22q13.33	Autism	+	–	+	+	–	–	–

*ASD* autism spectrum disorder, *ADHD* attention deficit and hyperactive disorder, *SQZ* schizophrenia, *BD* bipolar disorder, *OCD* obsessive–compulsive disorder, *A* anxiety, *GTS* Gilles de la Tourette syndrome

*Hahn and Friedman (1999); Lai et al. (2001); Ophoff et al. (2002); Moessner et al. (2007); Bauer et al. (2008); Djurovic et al. (2009); Pasmant et al. (2009); Walss-Bass et al. (2009); Wang et al. (2009); Weiss et al. (2009); Carroll et al. (2010); Elia et al. (2010); Gauthier et al. (2010); Maestrini et al. (2010); Pagnamenta et al. (2010); Rosenfeld et al. (2010); Wisniowiecka-Kowalnik et al. (2010); Girirajan et al. (2011); Kato et al. (2011); Levy et al. (2011); Ma et al. (2011); Spencer et al. (2011); Waga et al. (2011); Casey et al. (2012); Girirajan et al. (2012); Lamb et al. (2012); O’Roak et al. (2012); Prasad et al. (2012); Schaaf et al. (2012); Grayton et al. (2013); Myles-Worsley et al. (2013); Porton et al. (2013); Bacchelli et al. (2014); Gimelli et al. (2014); Noor et al. (2014); Schaaf (2014); Gillentine and Schaaf (2015); Nesbitt et al. (2015); Noor et al. (2015)

Our cohort has been clinically examined in great detail for psychiatric and behavioural disorders as well as a dysmorphological evaluation was performed by a clinical geneticist. Almost all patients in our cohort present mild cranial or facial dysmorphic features suggesting that having multiple mild dysmorphic features may be a clue to an underlying genetic cause, despite specific comparison was not possible. We suggest that adults with mild or moderate ID, psychiatric/behavioural disorders and mild dysmorphic signs are an especially CNV enriched group as shown in the present study.

We highlight there is a high familial burden of ID and neuropsychiatric disorders in all individuals with an inherited genetic cause mainly gathered in the pVOUS group. Inherited variants must be taken into account because they can act as susceptibility factors having an additive or synergistic effect (Pinto et al. 2010; Girirajan and Eichler 2010). The identification of a familial history in individuals with ID and neuropsychiatric disorders is challenging due to the continuous spectrum of the phenotype that could explain the discrepancy between family members. Therefore, pVOUS should be considered in larger studies to reinforce their pathogenicity for ID and co-morbid psychiatric disorders.

The data provided here from an adult cohort with mild-moderate ID and co-morbid psychiatric and behavioural disorders is essential to advance our knowledge of these pathologies and useful for genotype-phenotype correlations as well as contribute to the prognosis of the behavioural phenotype in children and adolescents with the same diagnoses. Most behaviours and organic/mental health problems are easier to work with and to understand when an aetiological diagnosis is delivered, which enables the planning of better medical intervention strategies. Furthermore, having a genetic diagnosis provides relevant information for families in terms of genetic counselling, allows improved care of all family members and provides an early diagnosis of related diseases, which is a significant issue to take into account when governments and authorities plan local and national health strategies. We propose that CMA testing together with a clinical genetics assessment would help to achieve more aetiological diagnoses in adult patients with ID and psychiatric disorders.

## Electronic supplementary material

Below is the link to the electronic supplementary material.

Additional file 1 Rare CNVs identified in the adult population affected by intellectual disability and co-morbid psychiatric/behavioural disorders. (Pat Id) Patient identification; (CNV, ISCN 2016) Copy number variant according to the International System for Human Cytogenetic Nomenclature edited in 2016; (bp) Base pair; (MAT) Maternal; (PAT) Paternal; (MAT and PAT) Inherited from both progenitors; (CGHa) Comparative genomic hybridization array; (BAC FISH) Bacterial artificial chromosome clones for fluorescent in situ hybridization; (MLPA) Multiplex ligation-dependent probe amplification; (MLPA autism) MRC-Holland Probemix P343; (MLPA subtel) MRC-Holland Probemix P036 and P070; (MS-MLPA) Methylation-specific MLPA with MRC-Holland Probemix ME028; (NA) Not analysed; (*) Polymorphic variant reported here due to its putative clinical relevance; (NP) Not present (XLSX 64 KB)

Additional file 2 Pathogenic CNVs and variants of unknown significance (VOUS) likely pathogenic (pVOUS): summary of data from literature (Coe et al., 2014 and DDD 2017) as well as public databases. (Pat Id) Patient identification; (chr) chromosome; (bp) base pair; (ISCN 2016) International System for Human Cytogenetic Nomenclature edited in 2016; (CNV) Copy number variant; (DD) Developmental delay (XLSX 34 KB)

Additional file 3 7q31 copy number variants reported in Database of Genomic Variants. (DGV) Database of Genomic Variants; (CNVR) Copy number variant region; (bp) base pair (XLSX 12 KB)
